# A friend in a forest of radiation-immune interactions: BAMBI improves antitumor effects by limiting radioresistance

**DOI:** 10.1172/JCI176061

**Published:** 2023-12-15

**Authors:** Sean Sachdev

**Affiliations:** Department of Radiation Oncology, Robert H. Lurie Comprehensive Cancer Center, Northwestern University Feinberg School of Medicine, Chicago, Illinois, USA.

## Abstract

Radiation therapy (RT) remains one of the most effective and utilized oncologic treatments available. While it can directly yield tumor cell death, its impact on the immune microenvironment is more complex, promoting either an antitumor response or, conversely, a tumor-promoting state. TGF-β, induced by RT, yields a more immunosuppressive environment, including potentially blunting response to immune-checkpoint blockade. In this issue of the *JCI*, Wang and colleagues demonstrate that RT reduced expression of bone morphogenetic protein and activin membrane-bound inhibitor (BAMBI), a TGF-β pseudoreceptor. Limiting this effect, or increasing BAMBI, improved RT-induced tumor cell killing, tumor response, and antitumor immune effects. This realization points to a pathway of potential clinical translation.

## Radiation’s impact on the tumor and immune microenvironment

Radiation therapy (RT) remains one of the most effective oncologic treatments available, whether used in a primary, adjunctive, or palliative fashion. Its predominant role in damaging tumor cells is well established: ionizing energy leads to irreversible DNA damage, mitotic catastrophe, and ultimately tumor cell death and tumor regression (1).

However, its impact on the tumor-immune microenvironment is poorly understood and more complex. While being able to induce inflammation within and around a targeted tumor, induced pathways can either favorably shift the milieu toward promoting antitumor immunity or, conversely, toward an oncoprogressive state driven by maladaptive mediators, such as myeloid-derived suppressor cells (MDSCs) or tumor-associated macrophages (TAMs), that ultimately promote radioresistance and/or tumor progression (2).

Over several years, better understanding of the immune microenvironment has led to important advances in transformative cancer treatment, such as immune-checkpoint blockade (ICB) therapy, which has improved outcomes in several cancer types, including melanoma, non–small cell lung cancer, and renal cell carcinoma (3–5). Despite its complex role in the tumor-immune microenvironment, in such a setting, RT offers the capability to focally induce inflammation and influence the tumor milieu. With continued study of immunomodulation via RT, a strategy of improved antitumor immunity remains promising.

## The role of TGF-β in tumor radioresistance

TGF-β is known to be induced after exposure to ionizing radiation and has been shown to be involved in long-term tissue injury and fibrosis (6). However, it also maintains a complex oncologic role; it may limit tumor growth during carcinogenesis, yet serve as a driver of cell proliferation and tumor progression in an established tumor state (7).

**Figure 1:**
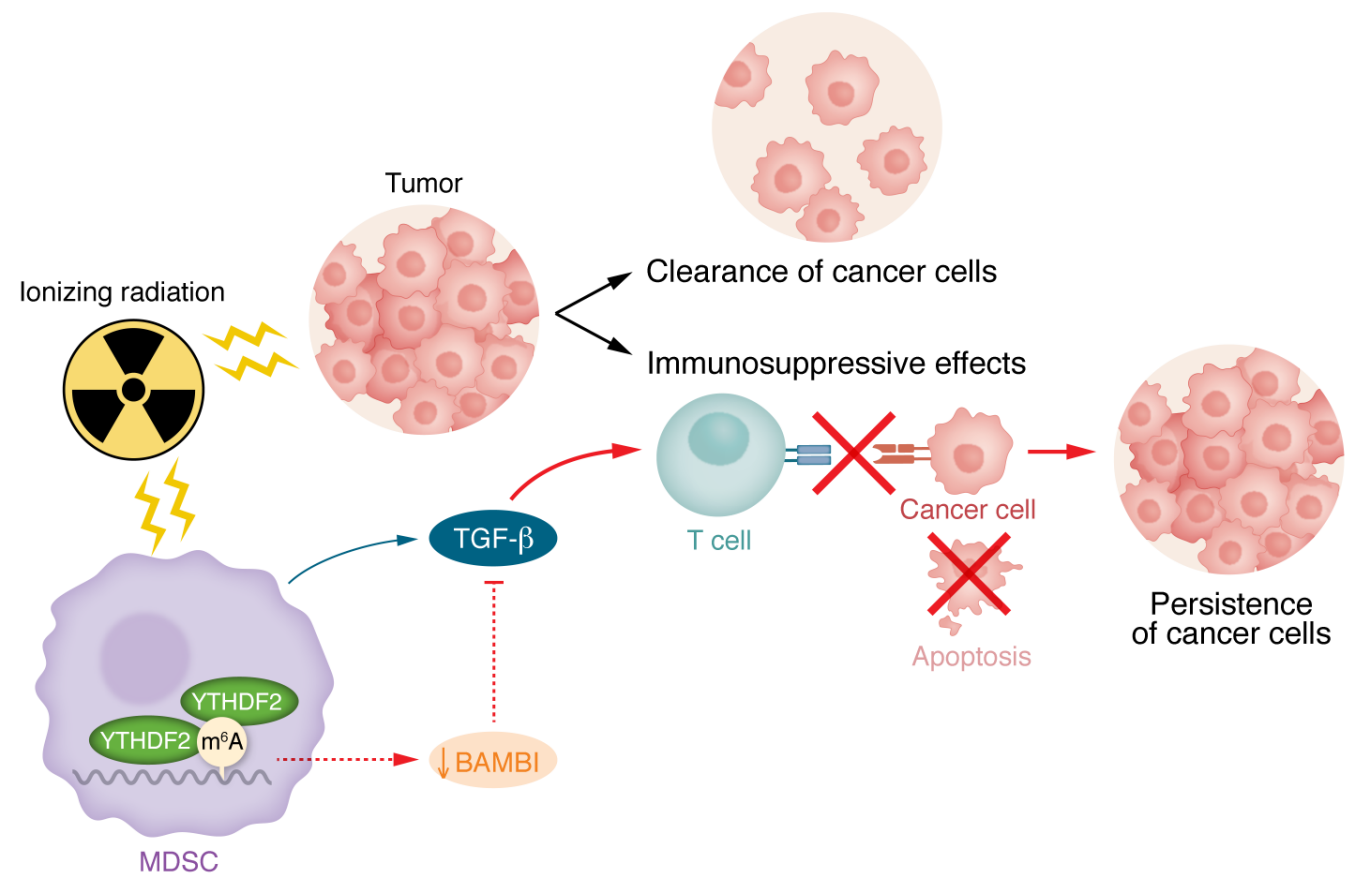
Targeting BAMBI during or after radiation treatment could improve tumor control. While RT can kill tumor cells, it also induces MDSCs to produce TGF-β, resulting in greater MDSC tumor infiltration, more T cell functional suppression, and greater tumor growth. BAMBI serves as a TGF-β pseudoreceptor and limits these effects. In irradiated MDSCs, interaction of YTDF2 results in the degradation of BAMBI mRNA. Following RT, therapeutic manipulation that increases BAMBI or limits its reduction may be key in improving tumor control. This strategy may be especially effective during an often-utilized combination of radiation and immunotherapy.

Over the years, more data have shed light on its negative impact on an established tumor-associated immune microenvironment. TGF-β has been shown, for example, to promote maladaptive macrophage infiltration (8) and induce a population of oncoprogressive tumor-associated neutrophils (TANs) (9). It has also been shown to be able to limit response to ICB (10). For patients undergoing tumor-directed RT, it appears that the negative consequences of TGF-β may be at least partly due to RT-induced reduction of the TGF-β pseudoreceptor, bone morphogenetic protein and activin membrane-bound inhibitor (BAMBI) (Figure 1).

## BAMBI functions as a TGF-β pseudoreceptor

In this issue of the *JCI*, Wang and colleagues (11) provide several lines of experimental proof to suggest that RT leads to YTHDF2-mediated m^6^A-dependent RNA degradation of *Bambi* in myeloid cells, particularly MDSCs. While similar in structure to TGF-β family receptors, by lacking an intracellular kinase domain, BAMBI is able to limit downstream effects (12). In essence, by serving as a pseudoreceptor, BAMBI is able to limit the negative effects of TGF-β after exposure to RT.

Wang and authors show that this reduction of BAMBI yielded greater MDSC tumor infiltration, more T cell functional suppression, and greater tumor growth. With overexpression of BAMBI, these changes were reversed, allowing improved tumor control. Importantly, in a variety of different tumor types, there appears to be a synergistic effect of BAMBI and RT in yielding enhanced antitumor response by CD8^+^ T cells and cytotoxic CD8^+^ T cells, overall tumor inhibition, and improved survival. In one tumor model, improved antitumor immunity through this combination also yielded distant tumor response (11).

Further, providing a crucial first link to an in-human study, the authors examined the impact of RT on BAMBI in tumor tissues from patients with lung cancer who were treated either sequentially or concurrently with ICB and high-dose RT (ClinicalTrials.gov NCT03223155). While the small quantity of patient samples limited generalizability, this type of clinical study is exactly what is needed to further investigate the role of BAMBI in being able to modulate the interplay between RT and antitumor immune response.

## The pathway ahead

These data point to BAMBI as a specific molecular target that could be pursued to improve local and distant tumor control during an often-utilized combination of radiation and immunotherapy (11). Other preclinical studies have examined a variety of potential avenues against TGF-β, including utilizing neutralizing antibodies or bifunctional inhibitory proteins that may limit this as well as other (i.e., PD-L1) pathways to improve antitumor immunity (13, 14).

While studies in humans have targeted TGF-β, several have been limited due to related adverse effects (15, 16). Perhaps a strategy that aimed to seek inhibition of the broader pathway elicited a higher probability of therapy-associated toxicity. Alternatively, a strategy that targets BAMBI offers a more specific pathway that could avoid negative unintended off-target effects. Since it has been shown to be directly influenced by RT exposure, future studies may seek specific interventions in this molecular pathway as a combinatorial strategy when RT is utilized.

Examination of stored tissue (from other prior prospective studies) that contain matched samples from before and after RT may offer confirmation for the findings in Wang et al. (11). Alternatively, future prospective trials could see this question posed as a tissue-centric transitional end point. If further evidence were able to confirm a strong link demonstrating RT’s effect on BAMBI, therapeutic strategies could be explored in either increasing BAMBI or limiting its reduction from RT. As a logical next step, such a strategy could also be employed with proven pathways of immunomodulation (i.e., using concurrent ICB). One may also begin to think about utilizing RT in tumors that have been historically thought to be immunologically “cold,” perhaps opening up frontiers for tumor types where utilization of ICB has not proven to be successful (17).

Modern highly conformal image-guided radiotherapy can produce high-dose gradients in any shape over high resolution (maximally sparing normal tissue) and is a backbone of multidisciplinary oncologic treatment. Many current regimens rely upon the synergy of well-tolerated systemic treatment delivered in conjunction with tumor-directed targeted conformal radiotherapy (18). If this microenvironment could be further modulated for improved potency and/or immunogenicity, it may not only improve initial tumor response, but also prolong the disease-free state through cell-mediated tumor immunity.

## Version 1 12/15/2023

Electronic publication
